# Test Rig for Investigating the Functional and Structural Fatigue of Shape Memory Alloy Wires Based on Different Activation Profiles

**DOI:** 10.3390/ma17061400

**Published:** 2024-03-19

**Authors:** Tobias Schmelter, Olivia Gawlik, Antonia Weirich, Bernd Kuhlenkötter

**Affiliations:** Chair of Production Systems, Ruhr-University of Bochum, Universitaetsstrasse 150, 44801 Bochum, Germany; olivia.gawlik@rub.de (O.G.); weirich@lps.rub.de (A.W.); kuhlenkoetter@lps.rub.de (B.K.)

**Keywords:** shape memory alloy, test rig, lifetime, activation profiles, fatigue behavior

## Abstract

This work presents a test rig developed for testing the lifetime of electrically and cyclically activated shape memory alloy wires. This test rig is developed to provide information on the functional and structural fatigue of the wires. Therefore, electrical activation on the test rig can be carried out using different activation profiles, because it is of great research interest to determine whether those profiles have a significant influence on the wire’s lifetime and functional behavior. The test rig monitors the process parameters such as stroke, current, voltage, and force. After presenting the electrical and mechanical design of the test rig, this publication evaluates an initial series of tests to demonstrate its functionality. Three different activation profiles are run in parallel on four identical test rig setups and are then evaluated. The functionality of the test rig is verified by a detailed evaluation of the process data on the one hand, and by comparing the results with existing literature on the other. The functionality of the test rig can thus be verified. At the same time, the strong influence of the different activation profiles on both the lifetime and the functional properties of the shape memory alloy wires becomes clear.

## 1. Introduction

Shape memory alloys (SMAs) have the unique function of autonomously returning to their original trained shape after a seemingly permanent, thus apparently plastic deformation by applying thermal energy [1,2]. If the material is restricted from transforming, useful forces can be generated [1,3]. Based on this effect, for example, actuators can be developed to substitute conventional drives, such as small motors, in an increasing number of application areas [4,5,6,7]. Furthermore, new application areas and automation potentials can be unlocked, especially in aerospace technology, based on the characteristic properties of SMAs [8,9]. For further implementation of the technology in more and more new and diverse application areas, the lifetime, and the understanding of the fatigue behavior/failure mechanisms of SMAs need to be further investigated. By investigating different electrical activation profiles, the authors aim to prove their influence on the fatigue behavior of SMA wires. This increases the reliability of SMA actuators, which remains a crucial disadvantage of SMA technology to this day [10,11], and therefore, increases their potential for commercial use. In further investigations, additional activation profiles can be compared in order to determine an optimum for the operation of SMA actuators. In this paper, a newly developed and constructed test rig is presented, which is used to investigate the activation behavior of SMA wires regarding their lifetime based on cyclic electrical activation. This test rig also offers the additional possibility of using different electrical activation profiles. Therefore, the wires are electrically activated by the internal resistance of the material, generating Joule heat. Subsequently, a first series of tests will be performed to evaluate the test rig and to investigate if using different activation profiles has any significant influence on the SMA wire’s lifetime and behavior.

## 2. Shape Memory Alloys

The following section briefly presents the fundamentals and associated principles of SMAs. Focus is placed on the fatigue behavior of electrically activated SMA actuator wires. The shape memory effect (SME) can occur in various alloys [2,3], whereby the most widely used alloy economically is a binary nickel–titanium alloy (NiTi), which is preferred over copper- and iron-based alloys due to its properties [1,2,11,12,13]. After the wire manufacturing process, the material does not have optimal shape memory properties [2]. The special properties are only imparted to the wire through subsequent thermomechanical treatment in combination with the alloy [11].

### 2.1. Phase Transformation of SMAs

From a materials science perspective, SMAs are classified as intelligent materials [1]. Due to their material properties, intelligent materials independently react to stimuli from the environment and adapt to those. An external stimulus, for example, an electrical impulse, triggers this effect [14,15]. When activated by thermal energy, SMAs undergo a change of shape along with generating a force when constrained [16,17]. This macro-logical shape change and force generation is due to a crystallographically reversible phase transformation of the metallurgical microstructure in the alloy [18,19]. This is a diffusionless transformation between two hard phases, where no change in chemical composition occurs [1,20]. In the low-temperature phase, the microstructure is martensitic, and in the high-temperature phase, austenitic [2].

**Figure 1:**
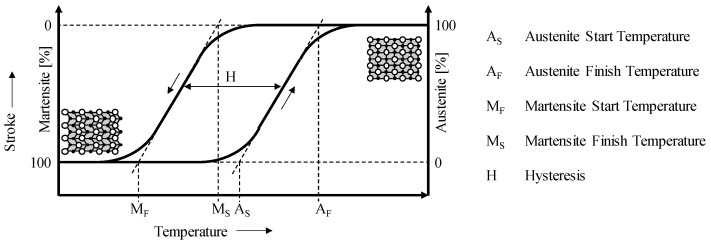
Hysteresis behavior of SMAs (own presentation) [1].

During activation/heating of the SMAs, a transformation from martensite to austenite occurs. The temperature at which austenite begins to form is known as the austenite start temperature (A_S_). When the phase transformation is completed and only austenite without residual martensite is present, the austenite finish temperature (A_F_) is reached. If the alloy is cooled, martensite formation starts at the martensite start temperature (M_S_) and is completed when the martensite finish temperature (M_F_) is reached. Martensite is stable at low temperatures (T < M_F_), and austenite at high temperatures (T > A_F_). Between A_S_ and M_S_, both phases are stable [21]. Due to internal friction during the phase transformation, temperature hysteresis occurs, so M_S_ ≠ A_F_ and M_F_ ≠ A_S_, resulting in four temperatures that must be defined to fully represent the temperature behavior of SMAs [18]. The relationship between these temperatures and the associated microstructure is shown in Figure 1.

### 2.2. Shape Memory Effects

**Figure 2:**
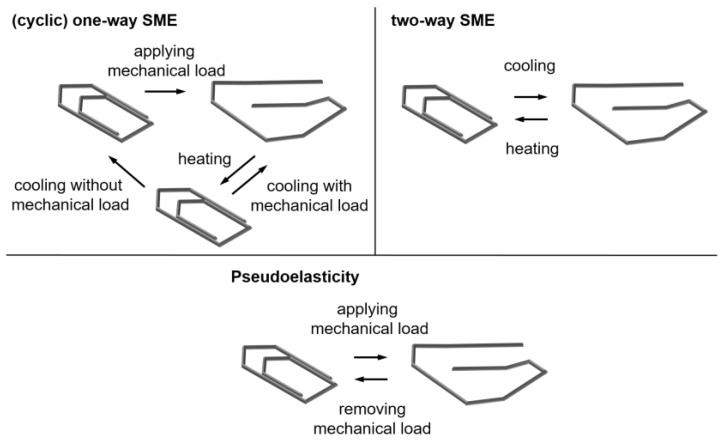
Pseudoplastic and pseudoelastic SMEs (own presentation) [23].

A distinction is made between three SMEs: the one-way effect, the two-way effect, and pseudoelasticity. The one-way and two-way effects belong to the thermally induced effects and thus to the effect of pseudoplasticity, while pseudoelasticity is a purely mechanically induced effect [1,2]. With pseudoelastic behavior, the material has the ability to withstand high strains at certain temperatures during a loading process and to autonomously restore its original shape when the load is removed [1,2,16,22]. In both of the effects associated with pseudoplasticity, a deformation remains even after the load has been removed and the original shape is regained by transformation [1]. Both effects are shown in Figure 2.

At the beginning of the process, a twinned martensite is present in the one-way effect, which is de-twinned by an external mechanical stress. As a result, the martensite undergoes plastic deformation [2]. These strains can be completely reversed [1]. In the case of NiTi, the possible reversible strains are around 8% [2]. If the austenite start temperature is exceeded during heating, the material begins to remember its original shape. As soon as the temperature ranges above the austenite finish temperature, the shape reversal is complete [16,24,25].

The two-way effect is divided into the intrinsic and the extrinsic two-way effect [26]. While the intrinsic effect results from the memorization of preferred variants through training in the form of thermomechanical training processes for high- and low-temperature phases, the extrinsic effect is primarily achieved by the fact that the one-way effect is used cyclically through a constantly applied external tension [2,26,27]. This external tension can be generated by a counter spring, an attached weight, or, in the case of an antagonistic design, also by a counter-running SMA wire [3,18,28,29]. During the thermomechanical training process, the material is deformed several times in the direction of the desired shape change, which serves to generate a preferred residual stress in the material [1,3]. Due to the low mechanical work in the intrinsic one-way effect, this effect is of secondary importance in technical applications and the field of actuator applications [2,20]. Thus, relevant for the test rig in this publication are wires that have the properties of the extrinsic two-way effect [2].

### 2.3. Fatigue Behavior

When SMA components are cyclically activated, fatigue behavior occurs after a certain number of cyclic activations, which contradicts the ideal image of fully reversible behavior—as it is often presented in theory [19,30]. Großmann defines fatigue of SMAs as a “change in material properties under cyclic (thermo)mechanical loading” [31]. How this fatigue behavior shows and when it occurs depends on a variety of factors, such as the quality of the material, which results from the microstructure generated in the manufacturing process, the environmental conditions, but also the load parameters in the application, and the resulting influences on the wire [26,32,33]. These parameters and influences include the force the wire needs to generate, the duration of activation, the selection of the electrical activation parameters, but also the stroke resulting from the activation. In most cases, fatigue cannot be attributed to a single cause, but is a multi-causal phenomenon [34].

Based on the different parameters and influences, fatigue in SMAs occurs due to an accumulation of lattice defects, which result in structural changes [35,36]. This leads not only to so-called structural fatigue in the form of crack initiation, crack growth, and fracture but also to functional fatigue [37]. Influencing factors can be, for example, the mechanical load [38], the melting [39] and manufacturing [40] process, the surface properties [41], or the ambient temperature [42]. In the case of functional fatigue, the material is no longer completely transformed back due to dislocations [43] or stabilized martensite variants [44,45]—or a combination of both—which have formed during cyclic activation [37].

In the case of structural failure, failure occurs through fracture or irreversible deformation, as with conventional metallic materials [46]. Due to the special properties of SMAs and their many interdependencies, however, various load parameters must be investigated individually in their respective application to make accurate lifetime predictions [2,30]. These parameters cause overstressing of the material, resulting in necking, formation of cracks, and ultimately fracture [47]. These macroscopic effects are caused at the microscopic level by an accumulation of microstructural defects under cyclic alternating stress, which is often favored by scratches or inclusions, as those lead to stress peaks in the surface [48].

Functional fatigue is often caused by high cyclic loading, which can be thermal or mechanical, and leads to a decrease or even loss of the functional properties of the SMAs [30,49]. In this process, new defects and dislocations are introduced into the crystal structure, or existing lattice defects are reoriented [30]. Furthermore, there is an obstruction of the internal interfacial movements, segregation of the phases involved in the transformation, the degree of order changes and relaxation and creep processes occur [2]. Due to various hotspots in the polycrystal, which lead to an inhomogeneous temperature distribution, fatigue is accelerated at individual points. Functional fatigue can be recognized not only by the reduced stroke or a change in hysteresis (accompanied by a change in phase-transformation temperature), but also by a change in conductivity and plateau stress [34]. Furthermore, functional fatigue of the SMA leads to a reduction of the effect amounts such as generated force or stroke, and a change of the material properties as well as a change of the martensitic form between the 1st and nth cycle [30,49,50,51]. Especially during the first activation cycles, in addition to the change in the microstructure, which occurs independently of the number of cycles for each activation of an SMA, a macroscopic change also appears, which represents a type of functional fatigue and is called the run-in effect [52]. This change in material behavior can be attributed to an accumulation of slip dislocations, which induce residual deformation, and the formation of stabilized martensite and dislocations [53,54].

### 2.4. Activation Profiles—State of the Art

There is already some preliminary work in the literature that deals with the use of different activation profiles. Zhang et al. 2023 and Vollach et al. 2016/Vollach et al. 2010 focus on the speed of activation [55,56,57]. However, only a single cycle is considered, and no lifetime study is carried out. In addition, only one activation profile (step response) is considered. Guan et al. 2021 combined short-term activation with a high electrical voltage and compared three different activation profiles [58]. Furthermore, a focus is placed on the resistance behavior, but the lifetime of the wires with regard to the individual activation profiles is not taken into account. There are also approaches to the activation behavior of wire bundles [59] or to the development of a model for predicting the electromechanical behavior of wires during electrical activation based on activation by a step response [60].

Fleczok et al. 2017 have already investigated different activation profiles of SMA wires regarding their lifetime [61]. However, the amount of energy applied to the different activation profiles was not kept constant and the force was not monitored throughout the tests. Nevertheless, the results of Fleczok et al. 2017 can be used to compare and confirm the plausibility of the tests generated in this work. A final comparison of the existing literature with the results generated by our new test rig in this work allows conclusions about the functionality of the new test rig and the equipment used to generate the activation profiles.

## 3. Design and Development of the Test Rig

To investigate functional and structural fatigue along with the associated fatigue behavior of electrically activated SMA wires, the conceptual design of a test rig is presented below. It is used to cyclically activate wires of different diameters until structural, fully functional, or even combined fatigue from functional-structural fatigue occurs. During the activation cycles, various sensors are used to record all relevant key figures and data to detect a change in the functional behavior of the wire, and thus allow conclusions regarding the influences affecting the fatigue. It is possible to vary the load parameters such as current flow, activation duration, activation profile, and the mechanical preload of the wire. Furthermore, the structure of the test rig is differentiated regarding the mechanical, sensory, and electrical components and described accordingly.

### 3.1. Requirements for the New Test Rig

The identification and definition of the requirements for the test rig development is based on Feldhusen et al [62]. The requirements are divided into three requirement categories (concept, product life phases, and planning) and assigned main features that must be fulfilled. As part of the idea generation process, the existing test benches in the research group and those presented in the literature were considered [63,64,65,66,67,68].

Some requirements are specified in advance, e.g., based on empirical values from other test benches; some other requirements are specified in detail later during the concept development phase. A reduced requirement list with only the elementary features is shown in Table 1. It serves as the basis for the development and construction of the test bench. The requirements are classified as D for demand for necessary specifications, and R for request for desirable but not critical requirements.

### 3.2. Final Mechanical Design

The test rig consists of four identical columns that are operated simultaneously. The electrical structure of the columns is decoupled, which means that each column can be controlled independently with specific parameters.

**Figure 3:**
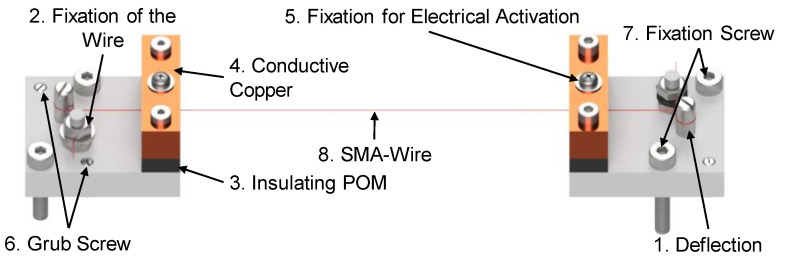
Device for clamping the wire.

To ensure that the tests are performed with high repeatability, the length of the wire to be tested must be the same for all tests. This is the only way to ensure that the different test series are comparable. For this purpose, the wire is placed in a clamping jaw at each end and clamped, whereby these clamping jaws are positioned at a predefined distance from each other. In the clamping jaws (s. Figure 3), the wire is deflected (Figure 3, Pos. 1), which prevents the wire from kinking at the clamping point and thus results in failure at this point. Direct clamping to the wire without this deflection could lead to damage when the mechanical preload is applied, resulting in the failure of the wire. Furthermore, the deflection ensures that the wire is guided in the center with repeatable accuracy. The resulting wrap friction ensures the best possible force absorption, which means that the downstream tensioning of the wire (Figure 3, Pos. 2) no longer has to absorb the entire force.

**Figure 4:**
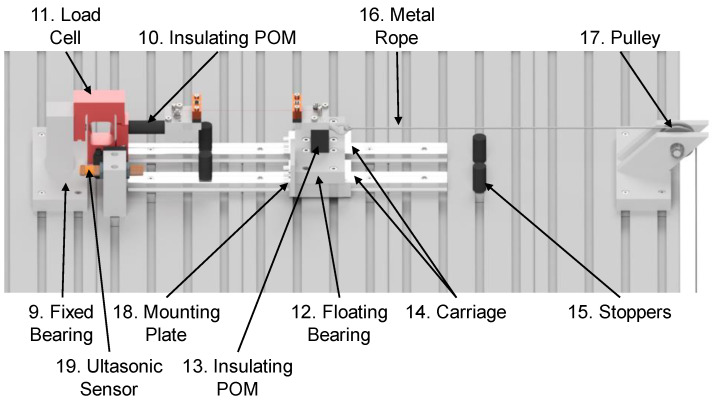
Mechanical design of the test rig.

The wire is then clamped between two plates, one made of the high-temperature-resistant polymer POM (Figure 3, Pos. 3) and one made from copper (Figure 3, Pos. 4). The upper copper plate is used for the electrical activation (Figure 3, Pos. 5) and applies the current to the wire, ensuring the best possible contact with the lowest possible loss. The lower plate made of POM serves as an insulator so that the current is dissipated completely through the wire and not into the test rig. In addition, the entire structure of the clamping device is connected to the test rig via POM (see Figure 4), which means that the current cannot be dissipated through the screws. Two grub screws (Figure 3, Pos. 6) and two cylinder head screws (Figure 3, Pos. 7) are used to place and connect the clamping jaws, including the wire (Figure 3, Pos. 8), to the test rig.

Figure 4 shows the further structure of the entire test rig. One clamping jaw is mounted as a fixed bearing (Figure 4, Pos. 9). The clamping is also separated from the rest of the test rig by a POM insulator (Figure 4, Pos. 10), which connects the clamping jaw to the load cell (Figure 4, Pos. 11). The entire fixed bearing is attached to a tabletop/grooved plate. The second clamping jaw is mounted on a floating bearing (Figure 4, Pos. 12), which enables compensation for the change in length of the wire during activation. Also insulated via POM (Figure 4, Pos. 13), the clamping jaw is mounted on a carriage (Figure 4, Pos. 14) sitting on a friction-minimized linear guide (drylin made by igus), which means that the influence of friction on the behavior of the wire can be neglected. Stoppers (Figure 4, Pos. 15) at the end of the linear guide prevent the carriage from sliding off the guide in case of failure of the wire. A metal rope (Figure 4, Pos. 16) is used to attach a weight, which can be freely adjusted, to the floating bearing and deflected via a pulley (Figure 4, Pos. 17). The weight force results in a mechanical pretension σ in the wire according to Formula (1), which is defined by the suspended weight *m* and the diameter or radius *r* of the wire.

(1) $$\sigma =\frac{F}{A}=\frac{m\cdot g}{\pi \cdot r^{2}}$$

A plate (Figure 4, Pos. 18) is attached to the floating bearing, onto which the ultrasonic sensor (Figure 4, Pos. 19) is aligned, and which thus reflects the ultrasonic waves of the sensor. This plate moves together with the floating bearing depending on the stroke generated by the wire and thus enables direct measurement of the wire properties. The ultrasonic sensor itself is attached to the tabletop.

Further hardware components required are a measuring amplifier (QuantumX MB 840 B made by Hottinger Brüel & Kjaer GmbH, Darmstadt, Germany), a laboratory power supply unit with arbitrary function (TOE 8805 made by TOELLNER Electronic Instrumente GmbH, Herdecke, Germany), and a measuring computer for data acquisition. The arbitrary function of the power supply units enables cyclical activation via programming activation profiles.

### 3.3. Sensor Technology

During the activation of the wire, as much relevant data regarding the fatigue behavior as possible should be recorded. The change in length generated per cycle (stroke), the force generated, and the electrical energy drawn by the wire, consisting of voltage and electrical current, are to be measured. Ultimately, further data can be calculated and derived from the data collected, such as the internal resistance of the wire or the mechanical stress in the wire. During testing, the sensors shown in Table 2 will continuously record data.

### 3.4. Electrical Design

All measured data from the test series are collected on the measuring computer, as this is where the data is recorded, saved, and displayed live for monitoring the tests. The measuring computer is connected to the measuring amplifier via LAN, where the various sensors converge. The measuring computer can also be used to control and program the power supply units (GPIB-USB-HS interface). Thus, it is possible to precisely define the duration of activation, the activation profile, and also the cooling time of the wire. The WaveControl software (Version Number: 0.8.5.0) from the power supply manufacturer Toellner is used for programming those activation profiles.

**Figure 5:**
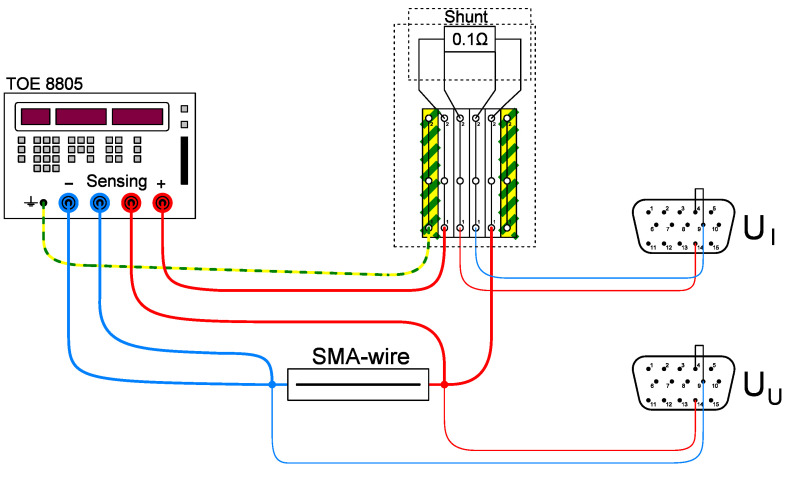
Electrical design of the test rig.

Two cables run from the power supply unit to the clamped sample. One line is used to conduct the current and activate the sample while the second one is used for sensing (s. Figure 5). Sensing enables a comparison between the voltage programmed and generated on the power supply unit and the voltage actually applied to the sample. Losses in the cables can lead to deviations between the actual and target voltage, which are detected and compensated for by sensing. To record the voltage applied to the sample, it can be tapped directly via a parallel circuit at the sample and recorded in the measuring amplifier (s. Figure 5, U_U_). As the current cannot be recorded as simply as the voltage, a shunt resistor (0.1 Ω) is installed in a series circuit. The voltage drop at this resistor can be used to determine the current (s. Figure 5, U_I_).

The current and voltage measurement cables, the load cell cable, and the ultrasonic sensor cable come together in a measuring amplifier.

### 3.5. Structure of Data Recording and Evaluation

The Catman software (Version Number: V5.5.3) from the manufacturer Hottinger Brüel & Kjaer GmbH, Darmstadt, Germany, is used for monitoring and data recording. The recorded data (stroke, current, voltage, and force) can be displayed and monitored in real time via the Catman user interface in the form of graphs and numerical values. In addition, a calculation channel is used to show which activation cycle the respective sample is in. For this purpose, the cycle counter is incremented on a rising edge of the current above 200 mA. The assignment of the cycles enables an exact evaluation in later analysis. All measured values are recorded at a frequency of 50 Hz.

## 4. Pre-Test: Adjustment of the Current Intensity to Evaluate 4% Stroke

Pseudoplastic SmartFlex wires from the manufacturer SAES Getters with a diameter of 0.353 mm were used for the tests, with the clamped length of the wires being 100 mm. The wires are made of a binary NiTi alloy. Before the actual long-term test is carried out, the desired current flow parameters must be determined. In this case, the target is to achieve an initial strain of 4% with an activation time of 2 s and a cooling time of 12 s. Only the current is determined via experiments. Based on the characteristic internal resistance of the wire, the electrical voltage is automatically adjusted according to Ohm’s law at a given current. Based on Formula (1), a weight of 3.45 Kg is attached to generate a mechanical preload of 350 MPa in the wire, which is below the manufacturer’s recommended maximum pretension of 400 MPa [69].

The manufacturer specifies a current of 3 A for a diameter of 0.4 mm and a current of 1.6 A for a diameter of 0.3 mm. Assuming linearity, a current of 2.3 A would be the result for the diameter used in this test. To check this specification, the current parameters for the main tests were determined in relation to the wire used in the tests by adjusting the current by 0.2 A every 250 cycles. The exact procedure for adjusting the amperage is shown in Table 3.

**Figure 6:**
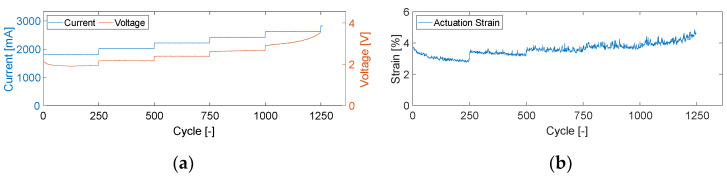
(**a**) Activation parameters and (**b**) strain over the cycles.

The course of the current and voltage over the entire 1500 cycles is shown in Figure 6 for column 1.

When looking at the strain achieved by the wire (s Figure 6b), at 1.8 A, 2.0 A, and 2.2 A (cycles 0–750), the required 4% strain is not constantly achieved and maintained over the 250 cycles. At a current of 2.6 A, on the other hand, the strain increases over 250 cycles, indicating that the current is too high, and the wire is damaged. A further examination of the stroke also shows that the wire elongates, which is another indication of damage to the wire. At 2.8 A, the wire fails after just a few cycles. This means that the requirement of a constant strain of 4% and an activation time of 2 s (cycles 751–1000) was achieved in the best possible way with a current of 2.4 A, resulting in these parameters being used for further test series. This result also corresponds to the manufacturer’s specifications.

## 5. Experiment Procedure: Variation of the Activation Profiles

**Figure 7:**
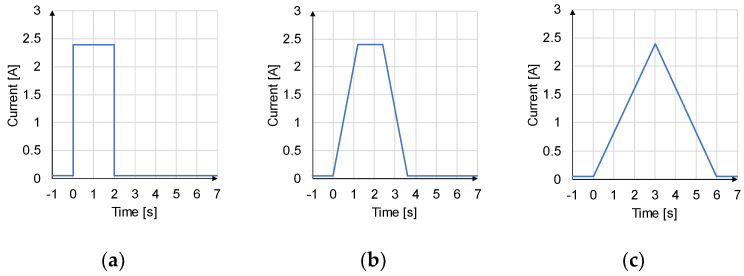
Comparison of the three different forms of activation: (**a**) step response, (**b**) trapezoidal form, and (**c**) triangular form.

The damage and fatigue behavior of SMAs is a multi-causal case, which is usually not attributable to a single, clearly definable influencing factor. Rather, it is often a combination of many individual factors, all of which contribute a small part to the failure and possibly reinforce each other. In the following, three different activation profiles and their respective influence on the lifetime of the SMA wires will be examined. This allows the test rig to be validated and the results serve as a preliminary investigation into whether different activation profiles have any significant influence on the lifetime and behavior of the wire at all. For better comparability of the results and the individual areas of the activation profiles, only profiles with a linear design were considered in this first investigation (as opposed to profiles with e.g., a sinusoidal curve). In most applications, the wire is activated via a step response (s. Figure 7a), whereby the current is switched on and off directly at 100%. It can be assumed that this rapid, abrupt activation results in an increased load on the wire. This behavior could have a negative effect on the lifetime of the wire, which is why different activation profiles with, for example, slower-rising edges are of interest.

For the comparability of the activation profiles, the electrical work *W* introduced must be the same for each profile. According to Formula (2), this results from the integral over time of the current *I* multiplied by the voltage *U*. It can be extended by Ohm’s law with the voltage *U*, resulting in the product of current *I* and the material’s resistance *R*. The resistance R depends on the material’s crystallographic structure and changes with phase transformation. The profiles are shown in Figure 7.

(2) $$W_{12}=\int_{t_{2}}^{t_{1}}U\cdot I\cdot dt=\int_{t_{2}}^{t_{1}}R\cdot I^{2}\cdot dt$$

The chosen strategy to keep the electrical work constant with different activation profiles is to adjust the activation duration while the maximum current of 2.4 A is kept constant. Three activation profiles are compared below: the step response (duration: 2 s), the trapezoidal shape (duration: 3.6 s), and the triangular shape (duration: 6 s). The resulting activation profiles are shown in Figure 7.

To perform the tests, all four test rig setups were operated simultaneously, and the same activation profile was used for each. After clamping the 100 mm long wire, the displacement sensor was calibrated and only then was the weight for pre-tensioning attached. In this way, the complete elongation of the wire (caused by the pretension and the activation of the wire) can be determined as part of the evaluation. All other test parameters are shown in Table 4.

## 6. Evaluation of Results

**Figure 8:**
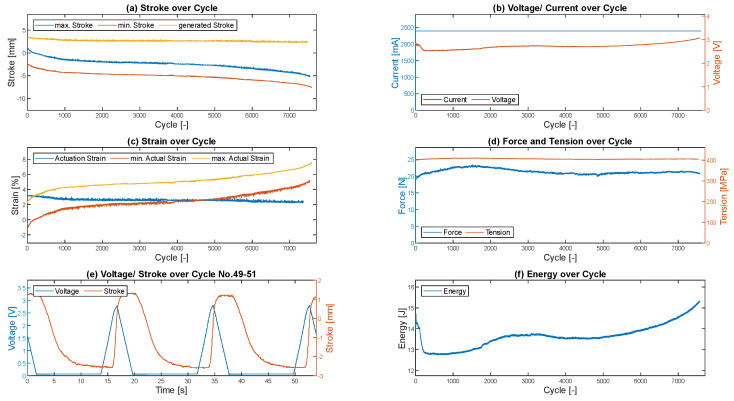
Overview of the activation parameters and recorded sensor data for monitoring the wire over its lifetime for an exemplary triangle activation.

To monitor the error-free performance of the tests, an overview is created for each tested wire. Figure 8 shows an example of the overview of the wire activated with a triangular form of the current on the second column of the test rig. The maximum and minimum values of the ultrasonic sensor per cycle are shown at the top left and the stroke generated in each case is determined by the difference between the two values. As the preload is applied by the attached weight, the wire elongates, causing the minimum stroke to fall into the negative range. This effect is further intensified over the lifetime by the fact that the wire becomes increasingly elongated due to the structural fatigue of the wire as a result of cyclic activation. Especially in the first 1000 cycles, a quite strong decrease in stroke can be seen, which is due to the run-in effect of the wire. This can also be seen in the strain (c), which is calculated from the original length of the wire (100 mm plus the change in length due to the application of the mechanical preload) and the generated stroke. The electrical voltage and stroke over time are shown for three exemplary cycles (e). The activation profile, which is determined by the current, can be seen in the graph of the electrical voltage. Based on the profile of the electrical voltage, it is not possible to recognize whether the wire has been fully activated compared to an activation using step response. However, based on the average stroke of 2.66 mm, it can be assumed that the energy applied is not sufficient to fully activate the wire and transform 100% of the microstructure.

To check whether the applied current is constant at the specified 2.4 A in all cycles, the current is plotted against the number of cycles (b), which is the case. Additionally, the electrical voltage is plotted over cycles, which decreases during the run-in effect and increases again over its lifetime. The measured maximum force applied by the wire per cycle (d) is added to the mechanical preload of 350 MPa, as this value represents the total load on the wire. Finally, the energy applied by the wire is calculated (f). Although the wire is activated with the same activation profile in all cycles, there are slight fluctuations in the energy absorption based on the damage mechanisms, such as elongation of the wire or a no longer fully reversible transformation between the martensitic and austenitic microstructure.

In addition to a detailed examination of the activation behavior over the individual cycles—which provides extensive insight into the damage behavior of SMA wires—the cycles achieved per activation profile are particularly important for evaluating the influence on the lifetime. Each test for each activation profile was carried out on each column of the test rig, i.e., four times in total, in order to detect possible fluctuations and outliers from individual tests. The number of cycles achieved for each activation profile is compared in Table 5.

**Figure 9:**
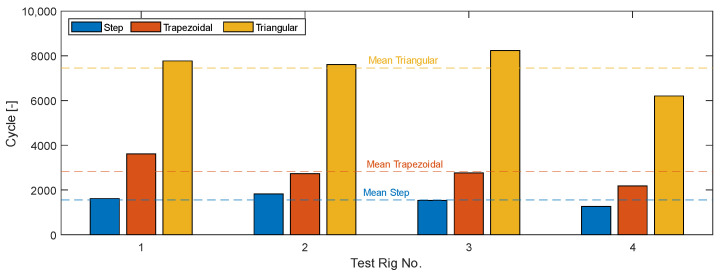
Comparison of the cycles achieved depending on the activation profile.

When looking at the cycles achieved, it becomes clear that the different activation profiles strongly influence the lifetime of the wires. The wires that were activated with a step response achieved the lowest average number of cycles at just 1556 cycles in average and were therefore the most likely to fail. The triangular profile achieved the highest number of cycles, and therefore the longest lifetime. Here, an average of just 7458 cycles was achieved in average, which is more than four times the lifetime of the step response. Figure 9 shows the results of the individual test rigs and the respective mean value of cycles achieved for each activation profile. It can also be shown that the four test stands are comparable, as the lifetimes achieved for each activation profile are similar on the columns. Small fluctuations in the lifetime are typical based on the different factors influencing the lifetime and differences in the microstructure resulting from production and handling.

However, a sole consideration of the cycles achieved is not meaningful at this point, as the activation duration has been adjusted to maintain the energy input. Therefore, the stroke achieved, the voltage absorbed, and the force generated by the wire must also be considered in evaluating whether the transformation was complete.

### 6.1. Stroke

The stroke generated at the respective test rigs is compared in Table 6. The average minimum and average maximum values of a column from all cycles as well as the average stroke generated per cycle are shown (compared to Figure 8a). In contrast to the lifetime exploited above, it can be seen that the highest stroke with an average of 3.549 mm was generated by the step response. This value is below the initial 4% elongation with a clamped length of 100 mm; this is because the 4% elongation was initially designed without the run-in effect of the wire. In addition, a degradation of the stroke occurs throughout the entire lifetime, because of functional fatigue, which means that the initial maximum stroke is no longer achieved. As the target is applying constant energy and not generating constant stroke, the current is not adjusted in this experiment and the reduced stroke is accepted. The two other activation profiles achieve even smaller strokes at 3.101 mm and 2.637 mm.

To simplify comparison and comparability, the values in Table 7 are compared in a standardized form, with 100% representing the maximum value achieved from all three activation profiles.

**Figure 10:**
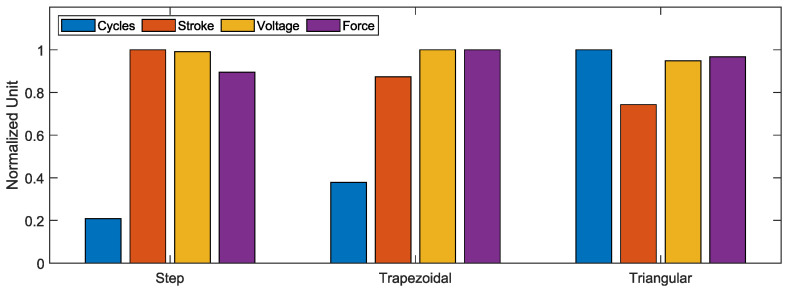
Comparison of all normalized sensor data.

In terms of the number of cycles, the step response only achieves 20.9% of the lifetime of the triangular profile, but the triangular profile still achieves 74.3% of the stroke that the step response achieved. The trapezoidal response also only reaches 37.8% of the lifetime, but still 87.4% of the original stroke. Thus, it can be seen that the decrease in lifetime is much greater as a function of activation than the decrease in stroke. The results are compared again graphically in Figure 10.

### 6.2. Voltage

Compared to the number of cycles and generated stroke, no trend can be seen in the electrical voltage, which is absorbed by the wire at a constant maximum current of 2.4 A. For step activation, an average voltage of 2.78 V is absorbed by the wire, for trapezoidal activation, an average voltage of 2.81 V, and for triangular activation, an average voltage of 2.66 V. It can hence be said that the electrical voltage for the step and trapezoidal profile is almost constant. With the triangular profile, the wire absorbs less electrical voltage. The curves are visualized in Figure 10. For a simplified overview, the values are normalized regarding the maximum value in each case, as already for the stroke and the number of cycles.

The increase in lifetime/achieved cycles over the activation profiles is greater than the decrease in the other three factors. However, it must always be noted that the original stroke of 4% is undercut in all cases.

### 6.3. Force

Figure 10 also shows the change in the average maximum force applied to the wire over the cycles achieved. As with the stroke and the electrical voltage, a drop in the force from the step response via the trapezoidal response to the triangular response can be seen.

**Figure 11:**
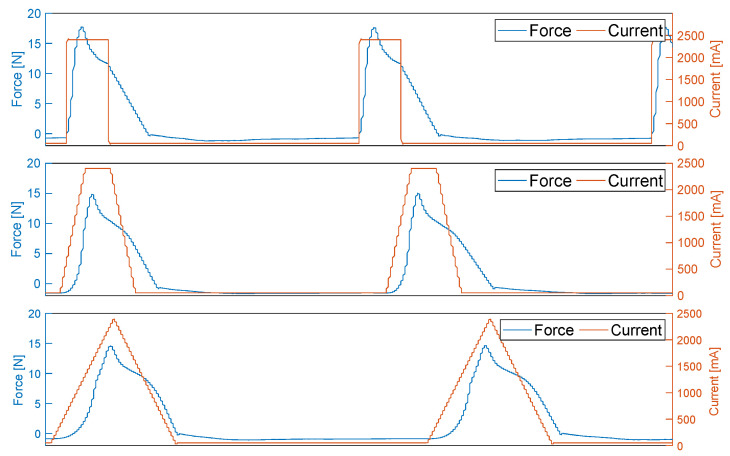
Comparison of the force curves depending on the activation profile at the 400th cycle.

At this point, the observation of a single cycle and the force curve over time as it results from the activation is also of interest. A rapid and abrupt change or application of force suggests that the lifetime of the wire is reduced by the jerky load. Figure 11 shows the force curves of each activation type at the 400th cycle together with the respective activation curve of the current strength—as recorded from the measured data. 

The force curve is similar for all three activation profiles, so that after an increase, the force slowly decreases during activation. After the end of activation, the force drops almost identically for all three profiles. In particular, attention should be paid to the area in the force curve in which the current is switched on and the subsequent increase. Although this looks similar for all profiles, it has different gradients and differences in the rise.

**Figure 12:**
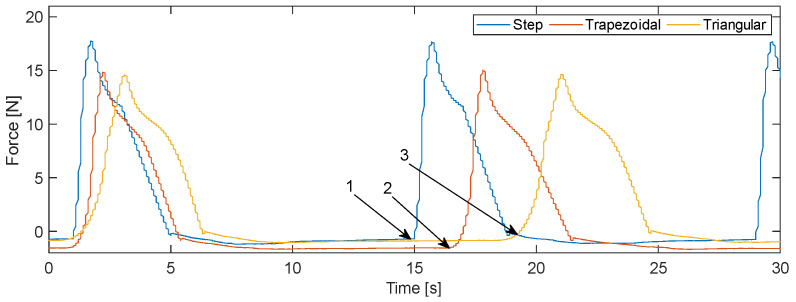
Superimposition of the force curves depending on the activation profile.

To illustrate the differences in the increase after activation, the force curves are superimposed once again in Figure 12. In the range from approx. 1 s to 3 s, the increase in force due to the activation of the wire can be seen. The step response exhibits the steepest rise and therefore applies the maximum force the fastest. With an offset of approx. 0.5 s, the force curve of the trapezoidal activation reaches its maximum, and with a further offset of approx. 0.5 s, the triangular response reaches its maximum value. In addition to the speed and associated gradient of the activation, the difference at 1 s, when the activation starts, is also clear. In the step response, the force increases immediately, resulting in an angle of almost 90° in the graph (Figure 12—1). The other two activation profiles produce a rather slow increase (Figure 12—2 & 3), which results in a more rounded shape of the graphs. The time offset indicates that not enough energy is introduced at the beginning of a rise that deviates from the step response to achieve A_S_. 

### 6.4. Comparison with Existing Literature on Activation Profiles

The influence of different activation parameters has already been considered in a paper by Fleczok et al. 2017 [61]. In their work, the activation duration of 1 s was kept constant, whereby there is no constant energy input, as the maximum current intensity is not adjusted depending on the profiles. The change in electrical voltage was also not considered and force monitoring was not included in the used test setup. Thus, the results are not directly comparable with the work at hand. Nevertheless, comparability can be recognized in terms of lifetime. The order from minimum to maximum lifetime as a dependency of the activation profile is the same in both, the tests in the literature and the tests in this work. The behavior regarding the stroke as a dependency of the activation profile is also comparable. Nevertheless, by using the measurement equipment shown here for the new test rig, it is possible to establish comparability between the activation profiles, which is not the case in the existing literature.

## 7. Conclusions

When summarizing the research results of this work, two different areas can be considered: the test rig and its structure itself as well as the influence of the different activation profiles on the lifetime of SMA wires.

### 7.1. Conclusion Concerning the Activation Profiles

Within this work, initial tests were performed regarding the fatigue behavior depending on different activation profiles. Three activation profiles were tested, and the results were compared with each other. The activation profiles show a clear difference in functional properties and lifetime of the SMA wires, with the triangular response having the longest lifetime and the lowest functional properties. This shows that activation profiles do influence the fatigue behavior of the wires. At this point, it is not yet possible to make a statement about the most suitable profile for the application with the maximum service life. However, it can be assumed that the triangular profile is the most suitable of the tested profiles because of the greatly increased service life with only minor losses in the range of stroke. Nevertheless, further tests must be performed in which other influencing factors such as the generated stroke are kept constant. Only then, a complete comparability of the profiles can be achieved. However, the differences will also be influenced by different activation times and the associated loss of energy through convection. The various influences of the activation profiles must therefore be investigated further and in depth. 

### 7.2. Conclusion on the Functional Testing of the Developed Test Rig

The test rig installed and evaluated in this work meets all requirements specified in the requirements list. The test rig generates repeatable and comparable results for the lifetime testing of SMA wires on the different columns, where small deviations in stroke and force can be seen between the individual columns. A pattern can be recognized, whereby a relationship between force and stroke becomes clear, as a higher stroke is generated with a lower force and vice versa. This suggests that the linear guides are not all optimally adjusted in the same way. The bearings on the linear guides can be adjusted in the X, Y, and Z directions using individual adjusting screws. The data recorded by the sensors do not show any deviations that would indicate faulty data recording. By using the sensing function, the actual required current value is applied to the wire and the characteristic resistance curve of the wire can be calculated by recording the voltage data, which can be used as proof of complete activation of the shape memory effect. Continuous force monitoring over the lifetime of the wire offers a variety of new approaches for a more precise investigation of the fatigue behavior of SMA wires. This will enable a deeper understanding of the dependencies and reinforcement between the functional and structural fatigue of SMA wires in the future.

## 8. Outlook

Regarding the different activation profiles, it seems promising to perform further investigations in the future, as the profiles differ significantly in terms of lifetime achieved, but also stroke and force applied. Different parameters should be kept constant and various comparisons should be made. 

In this first series of tests, the applied energy was kept constant, but this further influenced the behavior of the wires due to different activation durations and a resulting non-adiabatic system. Furthermore, the original stroke of 4% could be kept constant across all activation profiles by continuously adjusting the energization parameters. To do this, the losses due to convection would have to be determined and readjusted depending on an extended activation period. Keeping the activation time constant at 2 s—while adjusting the current—compared to the constant current of 2.4 A—while adjusting the activation time in this work—should also be investigated further by evaluating the different influences on the wire. Additionally, the current intensity could be set variably and the difference between the individual profiles due to convection with the ambient air would be reduced. Lastly, additional activation profiles can be applied and evaluated. This includes other activation profiles with a linear structure as well as sinusoidal activation profiles.

Looking more closely at the force curve over the lifetime would be of great interest in the context of the tests carried out. This has not yet been examined in depth in the existing literature and therefore offers the opportunity to develop new approaches for predicting fatigue and failure. Also, further conclusions could be drawn regarding fatigue and failure behavior. The wires used in these tests should also be used for downstream materials science investigations. Starting points for failure can possibly be derived from the fracture surfaces of the wires and, by combining the activation profiles with the manufacturing process optimized regarding the material properties, an optimized lifetime of the wires with better functional properties can be generated.

## Figures and Tables

**Table 1:** List of requirements for the developed test rig (extract of most relevant requirements).

Number	Type of Requirement	Requirement
***Concept***		
Material		
1.1	D	Nickel–titanium-based wires
1.2	D	Conductive material: copper
1.3	D	Insulating material: POM
1.6	D	Cooling medium: air
Geometry		
2.1	D	Clamping of wires with diameters up to 1 mm
2.7	D	Horizontal test bench setup
Mechanics		
3.1	D	Adjustable load up to 10 kg
3.3	D	Exploiting the wrap-around friction
3.8	D	Low-friction movement of the floating bearing
Energy		
4.1	D	Type of activation: Joule heating of the wire
4.2	D	Cooling through thermal convection and radiation
4.3	D	Option of switching the power supply on and off at timed intervals
Electrics/Electronics		
5.1	D	Min. power of the power supply unit: 50 W
5.4	D	Adjustability of an (infinite) number of cycles
Software		
6.2	D	Computer-aided programming of power supply units and activation profiles
Signal/Sensors		
7.1	D	Measurement of the voltage applied to the wire
7.2	D	Measurement of the current applied to the wire
7.3	D	Measurement of the force applied to the wire
7.4	D	Measurement of the stroke performed by the wire
7.5	R	Monitoring the ambient temperature
Safety		
8.1	D	Electrical insulation
8.4	D	Production of components by qualified personnel
8.5	D	Installation and connection of the electronics by qualified personnel
Ergonomics		
9.1	D	Performing hand movements at upper torso height at most
9.2	D	Performing hand movements at least at lower torso height
***Product life cycle phases***		
Assembly		
10.1	D	Mountability of the test setup
10.3	D	Can be dismantled and reassembled at any time if required
Usage		
11.2	D	For use at room temperature (20 °C)
***Organization***		
Planning		
12.1	D	End date of the construction: 30 June 2023
12.2	D	Investment costs as low as possible
Sustainability		
13.1	D	Maximum utilization of existing resources
13.2	D	Production of components in our own workshop

**Table 2:** Overview of sensors used in the test rig for data acquisition.

Sensor	Specification	Data Recording	Explanation
Ultrasonic sensor (UNAM 12U9914/S14 by Baumer)	Range: 20–200 mm Repeatability: 0.5 mm Resolution: <0.3 mm	Change in length of the wire (stroke)	The ultrasonic sensor continuously determines the length of the wire. The data can then be used to determine the stroke generated per activation cycle and the (overall) elongation of the wire. The change in stroke over the activation cycles can be used to investigate the functional fatigue behavior of the wires.
Load cell (S2M/500N by HBK)	Accuracy: 0.02% Force: <500 N	The force applied by the wire	Along with the change in the length of the wire, a force is generated, lifting an attached weight. To date, there have been no detailed studies on the force progression in SMA wires and resulting findings regarding the fatigue behavior.
Current sensor	Measured via: precision resistor Resistance: 0.1 Ω Tolerance: 0.5% Capacity: 10 W	Current applied to the wire	Both functional and structural fatigue result in a change in resistance in the wire due to elongation of the wire but are also based on the proportions of the martensitic and austenitic microstructure. As the resistance cannot be measured directly, it is calculated using the current and voltage (Ohm’s law).
Voltage sensor		Electrical voltage applied to the wire	see Current sensor
All measured values are combined and recorded in a QuantumX MX840B measuring amplifier with an accuracy class of 0.05% and a measuring rate of up to 40 kS/s			

**Table 3:** Data for evaluating the power supply parameters.

Start Cycle	End Cycle	Current [A]	Activation Duration [s]
0	250	1.8	2
251	500	2.0	2
501	750	2.2	2
751	1000	2.4	2
1001	1250	2.6	2
1251	1500	2.8	2

**Table 4:** Test parameters.

Test Parameter	Specification
Duration auf activation	2–6 s
Duration of cooling	12 s
Mechanical preload	350 MPa
Voltage	2.4 A
Diameter of wire	0.35 mm
Wire manufacturer	SAES Getters

**Table 5:** Cycles achived depending on the activation profile.

	Step	Trapezoidal	Triangular
**Rig 1 (cycles)**	1615	3615	7774
**Rig 2 (cycles)**	1821	2732	7613
**Rig 3 (cycles)**	1530	2763	8240
**Rig 4 (cycles)**	1261	2180	6205
**Average (cycles)**	1556.75	2822.5	7458

**Table 6:** Comparison of the generated stroke depending on the activation profile.

	Step			Trapezoidal			Triangular		
	Min [mm]	Max [mm]	Mean [mm]	Min [mm]	Max [mm]	Mean [mm]	Min [mm]	Max [mm]	Mean [mm]
**Rig 1**	3.286	4.179	3.621	3.003	4.121	3.361	2.653	3.754	2.996
**Rig 2**	3.198	4.364	3.540	2.7	4.038	3.075	2.26	3.437	2.657
**Rig 3**	3.473	4.678	3.898	2.847	4.425	3.364	2.43	3.928	2.968
**Rig 4**	2.735	4.020	3.136	2.213	3.81	2.605	1.574	2.615	1.927
**AVG**	3.173	4.310	3.549	2.691	4.099	3.101	2.229	3.433	2.637

**Table 7:** Standardized comparison of the generated stroke.

	Maximum	Step		Trapezoidal		Triangular	
**Lifetime**	7458	1556.75	20.9%	2822.5	37.8%	7458	100%
**Stroke**	3.549 mm	3.549 mm	100%	3.101 mm	87.4%	2.637 mm	74.3%

## Data Availability

Data are contained within the article.
